# Surface Properties of Ti6Al7Nb Alloy: Surface Free Energy and Bacteria Adhesion

**DOI:** 10.3390/jfb13010026

**Published:** 2022-03-07

**Authors:** Monika Krzywicka, Jolanta Szymańska, Szymon Tofil, Anna Malm, Agnieszka Grzegorczyk

**Affiliations:** 1 Department of Technology Fundamentals, University of Life Sciences, 20-612 Lublin, Poland; 2 Department of Comprehensive Pediatric and Adult Dentistry, Medical University of Lublin, 20-059 Lublin, Poland; szymanska.lublin@gmail.com; 3 Faculty of Mechatronics and Mechanical Engineering, Kielce University of Technology, 25-314 Kielce, Poland; tofil@tu.kielce.pl; 4 Chair and Department of Pharmaceutical Microbiology with Laboratory for Microbiological Diagnostics, Medical University of Lublin, 20-093 Lublin, Poland; anna.malm@umlub.pl (A.M.); agnieszka.grzegorczyk@umlub.pl (A.G.)

**Keywords:** titanium alloys, laser surface texturing, surface free energy, bacteria adhesion, biofilm

## Abstract

The laser micro-machining was carried out on a station equipped with a TruMicro 5325c laser emitting ultraviolet radiation (343 nm wavelength) in picosecond pulses. On the surface of the Ti6Al7Nb alloy, dimple texturing with a constant diameter of ~200 μm, different depths (from ~5 to ~78 μm) and density (from 10% to 50%) were produced. The value of surface free energy was determined with the Owens–Wendt method using two measuring liquids: distilled water and diodomethane. The Staphylococcus epidermidis strain was used to test the adhesion of bacteria. It was found that the surface free energy value is influenced by both of the texture parameters (density, depth). The density also affects the potential for biofilm formation. Based on the analysis, it was shown that with an increase in surface free energy, the number of adhering microorganisms increases exponentially. Moreover, the study shows that there is a correlation between the number of adhering microorganisms and surface free energy.

## 1. Introduction

Recently, the use of titanium and its alloys in medicine and dentistry has been rapidly increasing [1]. Titanium and its alloys, in comparison with other metallic materials, are characterized by the best biological inertness, even during a long service life. The properties of titanium and its alloys include, among others, high corrosion resistance, also in the body fluids environment. Titanium has the highest resistance to general, pitting and crevice corrosion as compared to other alloys used for implants. Due to their low density and good strength properties, titanium alloys are commonly used for joint replacements. In addition, titanium alloys are non-magnetic and have low electrical conductivity, which allows patients to undergo physical therapy procedures [2,3,4,5,6].

Septic loosening may be one of the reasons for the lack of stability of the joint replacements. Bacterial infection is initiated by the adhesion of bacteria to the implant surface, followed by bacterial colonization and biofilm formation [7]. The first stage of bacteria adhesion to the surface of a material is caused by physicochemical factors influenced, among others, by surface topography. The influence of surface topography on the phenomenon of bacterial adhesion is poorly known. An understanding of how surface properties affect bacterial adhesion helps modify the surface of biomaterials [8]. After biomaterials have been placed in the body, the process of osseointegration competes with that of bacterial adhesion to the bone tissue. Thus, the surface covered with bone tissue becomes less susceptible to bacterial colonization [9]. 

Mechanical methods used to change the surface topography, including machining, sandblasting, and chemical methods, such as oxidation, or acid etching, have disadvantages. These processes, used so far in dentistry and orthopedics, are characterized by a lack of selectivity, formation of undesirable impurities, causing changes in the structure and properties of the base material, and the inability to obtain repetitive textures [10]. Laser surface texturing is one of the methods of modifying titanium biomaterials used to improve their properties [5,11]. This technique enables a precise, repeatable change in the topography of almost all solid materials without damaging the surface [5,12]. The use of laser micromachining helps avoid the formation of impurities, it is easier to control and more precise; also, the heat-affected zone is reduced, and a small, concentric deposition zone of the vaporized material is created around the individual texture elements [8,13,14,15,16,17]. Laser surface texturing has great potential for functionalizing the surface of biomaterials, because it allows the use of a wide range of materials on the surface, which can be reproducibly and quickly produced in various shapes and sizes of textures [10,18,19,20]. Laser surface texturing is one of the few eco-friendly and cost-effective approaches used for texturing at the micro and nano level [21]. Laser surface texturing is a promising method to tailor wettability, surface energy, surface topography, and morphology through which cell attachment and proliferation can be modulated [21,22]. Moreover, ultrashort pulsed laser treatment is a good candidate for the production of man-made antibacterial surfaces as it can be used to produce textures whose sizes are lower than bacterial size [23].

In line with our knowledge, the effect of laser texturing of titanium alloy surface on selected properties was investigated, but no detailed analysis of the correlation between texture parameters, surface free energy, and bacterial adhesion has been carried out. Therefore, the novelty of our research is the analysis of the relationship between the texture parameters and selected properties of the titanium alloy subjected to laser surface texturing. The aim of this article is to analyze the influence of the selected parameters (density, depth) of the laser surface texturing of the Ti6Al7Nb alloy on the value of surface free energy and on the quantity of adhering microorganisms, as well as on biofilm formation. 

## 2. Materials and Methods

### 2.1. Laser Surface Texture Preparation

The samples made of Ti6Al7Nb titanium alloy were the subject of the research. The chemical composition of the alloy complied with ISO 5832-11 and was as follows: Fe max. 0.25%, O max. 0.2%, N max. 0.05%, C max. 0.08%, H max. 0.009%, Al 5.5–6.5%, Nb 6.5–7.5, Ta max. 0.5%, and the rest of Ti. The surface roughness (R_a_) of the control (non-textured) and experimental (textured) samples was 0.16 µm, for the determination of surface free energy (SFE), and 0.06 µm, for the evaluation of biofilm formation and the quantity of adhering microorganisms. The laser micro-machining of the surface layer of cylindrical samples was carried out on a bench equipped with a TRUMPF TruMicro 5235c laser with the following characteristics:

– laser type: diode-pumped pulse disk laser with harmonic generation 3;
– wavelength: 343 nm;
– average power: 5 W;
– pulse duration: 6.2 ps;
– 400 kHz pulse frequency with the possibility of dividing by a number from 1 to 10,000;
– maximum pulse energy: 12.6 μJ;
– fluence: 4.8 J/cm^2^.

On the surface of the samples, dimples with the constant diameter (~200 μm), different depths (from ~5 to ~78 μm), and different density (from 10% to 50%) were produced. In order to determine SFE, dimples with the following parameters were produced:

– 15.86% density, 15.69 μm depth;
– 44.14% density, 15.69 μm depth;
– 10.00% density, 41.50 μm depth;
– 30.00% density, 5.00 μm depth;
– 15.86% density, 67.31 μm depth;
– 30.00% density, 41.50 μm depth;
– 30.00% density, 78.00 μm depth;
– 44.14% density, 67.31 μm depth;
– 50.00% density, 41.50 μm depth.

In order to assess the biofilm formation and determine the number of adhering microorganisms (CFU/mL), dimples with the following parameters were produced:

– 50% density, 5 μm depth;
– 10% density, 5 μm depth;
– 10% density, 78 μm depth.

The conducted tests have shown that, for the Ti6Al7Nb alloy, the best method is to use laser texturing in an argon protection, at 100% power and the surface scanning speed with a laser beam of 50 mm/s [24]. After scanning the surface twice with a laser beam with the frequency of 80 kHz, individual textured elements with the depth of approx. 5 μm were obtained. Greater depths were obtained using the 100 kHz frequency, and its multiples, of laser beam scanning from 5 to 35 times. The texture microgeometry parameters were analyzed using the HIROX KH-8700 digital microscope (Hirox, Tokyo, Japan). After laser texturing, the samples were bathed in distilled water at 55 °C in an ultrasonic cleaner (Ultron, Dywity, Poland) for 10 min.

### 2.2. Surface Free Energy Determination

The tests were carried out with the Owens–Wendt method using two measuring liquids: distilled water and diiodomethane. The following values of SFE constants of measuring liquids and its components—polar and dispersive were assumed: γ_w_ = 72.8 [mJ/m^2^], γ^p^_w_ = 51.0 [mJ/m^2^], γ^d^_w_ = 21.8 [mJ/m^2^], γ_d_ = 50.8 [mJ/m^2^], γ^p^_d_ = 2.3 [mJ/m^2^], γ^d^_d_ = 48.5 [mJ/m^2^]. Prior to testing, the samples were washed three times with Loctite 7063. Drops of measuring liquids with a constant volume of 4 μL were applied automatically by the PGX-2 goniometer mechanism. The contact angle measurements for both water and diiodomethane were based on the measurement of ten drops. The obtained values of the angles were averaged and the SFE value was calculated on this basis. Three samples with the same texture parameters were prepared and three measurements were taken into account for averages. In order to observe the drop and to measure the contact angle, a stereoscopic microscope together with a camera and MicroScan version 1.3 software (Omron Automation, Kyoto, Japan) were used.

### 2.3. Bacterial Cell Culture and Assessment of Bacterial Adhesion and Biofilm Formation

A Staphylococcus epidermidis reference strain from the American Type Culture Collection (ATCC): Staphylococcus epidermidis ATCC 12228 was used.

From a fresh 24-h solid culture of Staphylococcus epidermidis ATCC 12228, an inoculum was prepared with a density of 0.5 on the McFarland scale (0.5 on the McF scale = 1.5 × 10^8^ CFU/mL, CFU—colony forming units). For this purpose, a small amount of microorganism was transferred with a sterile oese from the solid tryptic soy medium (TSA) and suspended in sterile, tryptic soy medium (TSB). After thorough mixing of the suspension, measurements were made using a densitometer (Densimat ATB 1550, bioMerieux, Florence, Italy).

The prepared bacterial inoculum was collected and transferred into wells on sixteen-well Nunclon ™ titration plates with TSB medium, resulting in a starting suspension density of 1.5 × 10^7^ CFU/mL. In the medium with staphylococci suspension, samples were placed, each in a separate well. This was incubated under aerobic conditions at 35 °C for 24 h (biofilm formation period) and another 24 h (creation of a mature biofilm). After this time, samples were rinsed with 3 mL of sterile, buffered saline without calcium and magnesium (PBS) and then placed in fresh TSB medium. After incubation, samples were removed, rinsed again and placed in 3 mL PBS, in tubes, and shaken for 30 min at 1000 rpm. Then dilutions were made from 10^−1^ to 10^−10^, plated on TSA medium and incubated at 37 °C for 24 h. After incubation, the colonies were counted (Counter Colony Scan 1200, Interscience, Saint Nom, France) and converted into CFU/mL. At the same time, substrate control and viability control for microorganisms were carried out.

At the same time, a second test was conducted in which samples of 3 mL PBS were rinsed after incubation, placed in test tubes, 1% crystal violet was added and stained for 3 min (time required for coloring of *S. epidermidis* biofilm formed on the surface of titanium samples). The samples were then washed with 3 mL of PBS, placed in test-tubes and shaken in 1 mL of 96%—analytical grade ethyl alcohol for 10 min at 1000 rpm. After this time, the crystal violet washed out from the staphylococci biofilm was collected and added to the 96-well plates to read the *OD* (optical density) in the ELX 800 reader (Bio-Tek Instruments, Inc., Winooski, VT, USA) at 570 nm wavelength. The KC Junior for Windows program (Bio-Tek Instruments, Inc.) was used in the readings. All activities were performed under aseptic conditions. The obtained results are the average of four measurements.

### 2.4. Statistical Analysis

Using Dell Statistica v. 13.1 (Dell Inc., Cracow, Poland, 2016), the analysis of interdependencies between the variables was performed. In the first step, it was examined whether there was a correlation between the independent variables (density, depth) and the dependent variables (SFE, CFU/mL, *OD*). After establishing that a correlation between the studied variables occurs, a regression function was found. The goal of multiple regression is to quantify the relationships between the independent variables and the dependent variable. The next step in the analysis of interdependencies was the verification of the model, which consisted in checking whether the assumptions of the model were met: the significance of linear regression, the significance of partial regression coefficients, no collinearity (redundancy) between independent variables, the assumption of homoscedasticity, which means that the variance of the random component (the residual ε_i_) is the same for all observations, no autocorrelation of residuals, normal distribution of residuals, and the random component (residual ε_i_) having the expected value equal to 0. Then, 3D surface charts/scatterplots were made. The data were considered significant at *p* < 0.1. The results of the SFE were analyzed of variance (ANOVA). The significance of differences between the assessed mean values was analyzed with Tukey’s test at the significance level *p* < 0.1.

## 3. Results

### 3.1. Topography of the Laser Textured Surfaces

**Figure 1:**
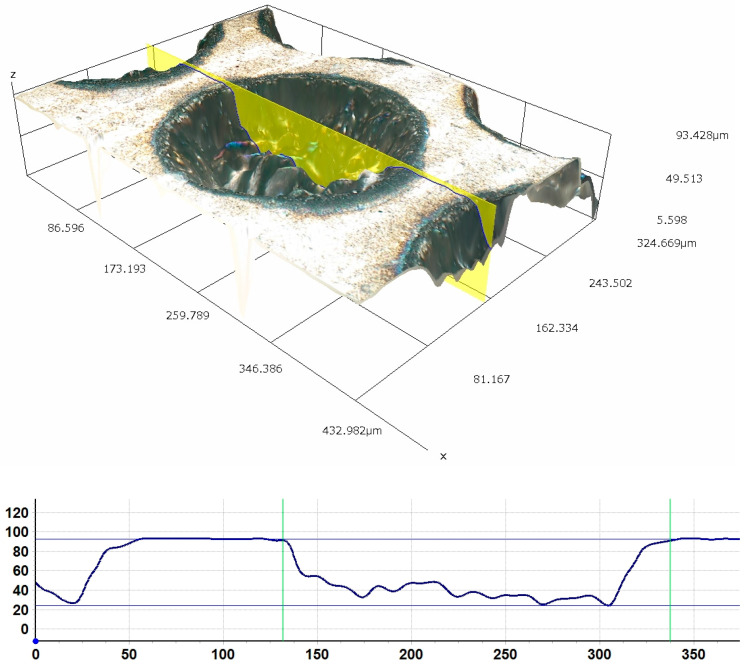
Profile and image of a single texture element with the diameter of 205.52 μm and the depth of 68.57 μm. Experiment parameters: frequency 100 kHz, laser beam scanning multiple 31.

**Figure 2:**
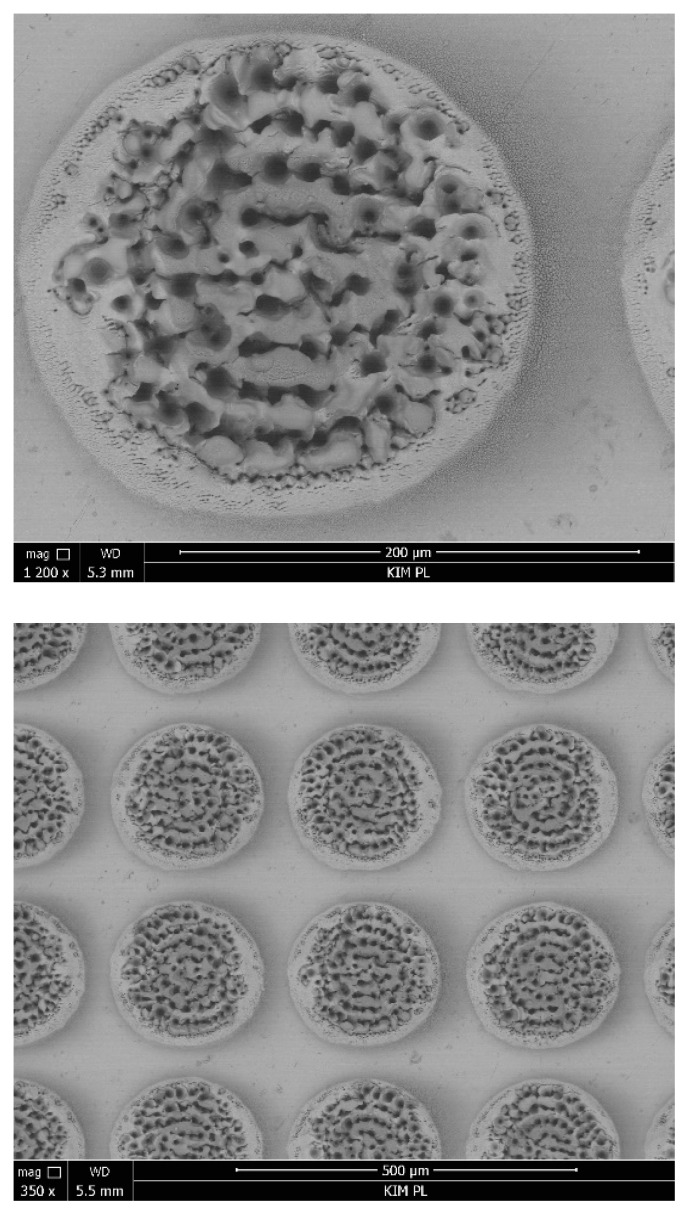
SEM images of the surface with texture (depth ~5%, density 50%).

In order to assess the quality of the produced dimples, their profile was examined. Figure 1 shows the profile and image of a single texture element with the diameter of 205.52 μm and the depth of 68.57 μm. The difference between the depth of the dimples according to the experiment plan and the depths obtained is up to 7.5%. Figure 2 shows SEM images of the texture with the depth of ~5% and the density of 50% at different magnifications (350× and 1200×).

### 3.2. Surface Free Energy Determination

For the reference sample with R_a_ = 0.16 μm, the SFE value is 46.5 mJ/m^2^. Table 1 shows the SFE values for individual samples. The highest SFE value, i.e., 58.6 mJ/m^2^, was recorded for samples whose surface dimples with 50% density and the depth of 41.5 μm were produced. The lowest SFE value, i.e., 42.3 mJ/m^2^, was recorded for samples whose surface dimples with the density of 15.86% density and the depth of 15.69 μm were produced; the SFE value was lower than for the non-textured sample. The difference between the lowest and the highest value is 16.3 mJ/m^2^ (38.5%). The analysis of variance showed that the mean SFE values for the tested samples are not identical. In the Tukey test, four groups were distinguished according to the mean SFE values. As the results show, the mean SFE values for samples with the depth of 15.69 μm do not differ significantly. This also applies to samples with the depth of 67.31 μm.

### 3.3. Bacterial Adhesion and Biofilm Formation

For the reference sample with R_a_ = 0.06 μm, the *OD* value was 0.171 and the number of CFU/mL was 6.73 × 10^5^. Table 2 shows the number of CFU/mL *S. epidermidis* ATCC 12228 biofilm forming on individual titanium alloy samples after laser surface texturing.

*S. epidermidis* ATCC 12228 showed the greatest adhesion to the surface on which the dimples with the highest density were produced. Comparing the adhesion to the surface on which the dimples with the same density and different depths were produced, it can be seen that a significantly higher value of CFU/mL was noted for a greater depth of dimples. The difference between the lowest and the highest value is 3.43 × 10^6^ (163.3%). 

The highest *OD* value, which reflects the potential for biofilm formation, was noted for the surface on which the dimples with the greatest density were produced. Comparing the *OD* values for samples on which the dimples with the same density and different depths were produced, it can be seen that a higher value was noted for the smaller depth of the dimples. The difference between the lowest and the highest value is 0.12 (37.9%).

## 4. Discussion

The multiple regression analysis for the SFE dependent variable shows that the coefficient of determination is 0.7, which means that the model explains 70% of the variability. The linear correlation coefficient R is equal to 0.84 and indicates that there is a very high correlation between the dependent variable and the independent variables. The regression coefficients for the variable density and depth are important. The standard error of the intercept parameter in relation to its value is relatively small. The linearity is checked by the F test. The *p*-value for this test is 0.0025, i.e., the regression equation is significant. In order to verify the model, a redundancy test was also carried out. The tolerance for all variables is high. There was no collinearity (redundancy) between the independent variables. It has been established that the assumption of homoscedasticity is fulfilled. To see if there was any autocorrelation of the residuals, Durbin–Watson statistical analysis was carried out. The value of statistic d is 0.85, which means that there is the autocorrelation of residuals. This may be due to the small amount of data (9) or the influence of other factors on the SFE value. It was assumed that the residual distribution is a normal distribution. The random component ε_i_ has the expected value equal to 0. Based on the result sheet, the mean value of the residuals is equal to 0. For the analysis of the residuals, a measure of the Cook’s distance was performed. These measures allow for determining whether a given observation can be included in outliers, while distance analysis makes it possible to conclude that none of the cases has a significant impact on the regression equation.

Based on the analysis of the residuals and the correlation coefficient and determination, it can be assumed that the model fit to the empirical data is correct.

The regression equation is:

(1) SFE =39.71+0.22× density +0.10×depth ± 2.28

**Figure 3:**
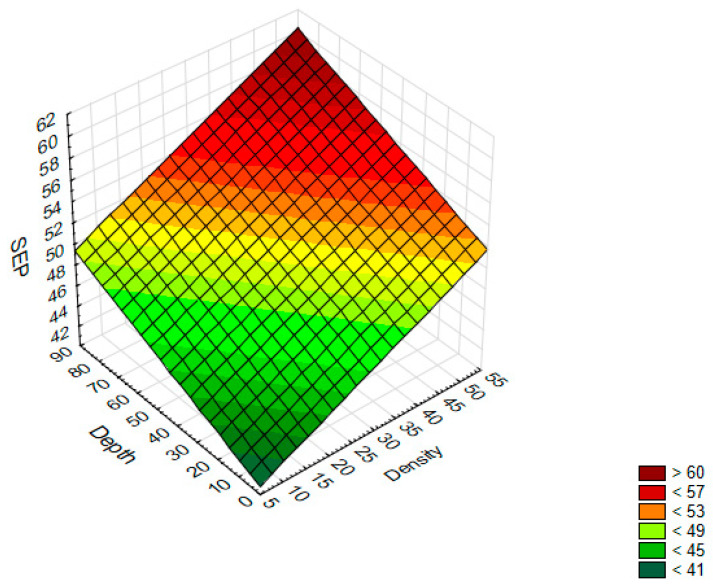
A 3D surface chart—SFE with respect to the density and depth of a single texture element.

This means that if the density increases by 1%, the SFE value will increase by 0.22 mJ/m^2^. However, if the depth of a single texture element increases by 1 μm, the SFE value will increase by 0.1 mJ/m^2^. The data and the surface suited to them are shown in Figure 3.

Based on the graph, it can be concluded that higher SFE values can be obtained for samples on which the dimples with a high density and high depth were produced.

Few researchers have studied the effect of microtextures on wetting systems. Jain et al. [25] examined conical dimple textures of different densities on Ti6Al4V fabricated by micro-milling and they showed that SFE values changed for samples of different densities. Wang i in. [26] found that the cylindrical surface reduced the contact angle and accelerated the spreading process. Pfleging and Kumari [5] showed that higher SFE is observed on the surface on which dimple texturing was made than on a linear texturing surface. Sęk and Antoszewski [27] indicated that a parameter constituting the mutual distance of the edges of microtexture had a significant impact on the SFE value. As this distance increases, the value of SFE increases. 

In this work, it has been established that there is no correlation (*p* > 0.1) between the independent variables: density and depth of a single element of texture, and the dependent variable CFU/mL. The independent variable: density, is correlated (*p* < 0.1) with the dependent variable *OD*. The standard error of the intercept parameter in relation to its value is relatively small. The coefficient of determination is equal to 0.98, which means that the model explains 98% of *OD* variation. The linear correlation coefficient R is equal to 0.99, and means that there is almost a full linear relationship between the dependent variable and the independent variable. The linearity is checked by the F test. The p level for this test is 0.09, i.e., the regression equation is significant. It was assumed that the residual distribution is a normal distribution.

The regression equation is:

(2) OD=0.3+0.003× density ± 0.01 

This means that if the density increases by 1%, the *OD* value will increase by 0.003.

The verification of the model showed that the assumptions of the randomness of deviations and of the lack of autocorrelation of the residuals are not met. This may be due to the small number of data (3) or to the influence of other factors on the *OD* value.

To the best of our knowledge, only Jain et al. [25]. have studied the effect of texture density on bacterial adhesion. Jain et al. [25] showed that cell densities are different for samples with different density of textures. Xu and Siedlecki [9] indicated that the size, spacing and shape of the produced surface features play a significant role in cell adhesion. Hsu et al. [28] indicate that no universal dependence between the topography of the surface and the number of attached cells was observed. The modification of surface features can be carried out to control the degree of bacterial adhesion [29]. Rajab et al. [8] produced hybrid hierarchical surface structures on Ti6Al4V using picosecond laser texturing to achieve anti-biofouling characteristics. Capitanu et al. [30] produced a spike structure on Ti6Al4V and the results showed that this structure could not provide the antibacterial effect. Lutey et al. [31] showed that laser-induced periodic surface structures produced at a laser wavelength of 1030 nm reduced Escherichia coli (*E. coli*) retention by over 99% and Staphylococcus aureus (*S. aureus*) by over 80% compared to the reference samples. Suraj Nanduru et al. [21] analyzed square and triangular textures. The width and depth of the square pit pattern and triangular pit pattern profiles are 100 μm and 90 μm, respectively, and contact angle of 134° (square pit) and 112° (triangular pit). The texture density of the square pit pattern and triangular pit patterns are 16% and 8%, respectively. Among the textures used in research, the square pit pattern showed a higher contact angle and better biofilm inhibition as compared to the triangular pit pattern. Available literature reports indicate that superhydrophobic surfaces reduce the adherence of bacteria [32,33,34]. Ahn et al. [35] mentioned that bacteria strains with high SFE preferably adhered to surfaces with the high SFE, while bacteria strains with low SFE adhered to surfaces with lower SFE.

The change in surface roughness and topography influences the change in SFE [36], and thus, also, affects the interaction between the bacteria and the surface of the biomaterial, and then the development of the biofilm [9]. High SFE increases the interaction between the surface of the implant and the biological environment, the spread of cells, and their adhesion [1,37,38,39]. Surface Free Energy can be used to assess the degree of interaction between the material and the organism [10]. Using formula 1, the SFE value was calculated for the following texture parameters:

– 50% density, 5 μm depth;
– 10% density, 5 μm depth;
– 10% density, 78 μm depth.

An analysis of the correlation between the CFU/mL and *OD* variables, and SFE was carried out. There was no correlation between *OD* and SFE. After determining that there was a correlation between the quantity of adhering microorganisms (CFU/mL) and SFE (*p* > 0.1), the regression function was found. The exponential function has been adjusted to the empirical data. The regression equation and regression coefficients are significant. The correlation coefficient R = 0.996, indicates a very good fit. The regression equation is:

(3) CFU/mL =19967.7× exp(0.1× SFE)

**Figure 4:**
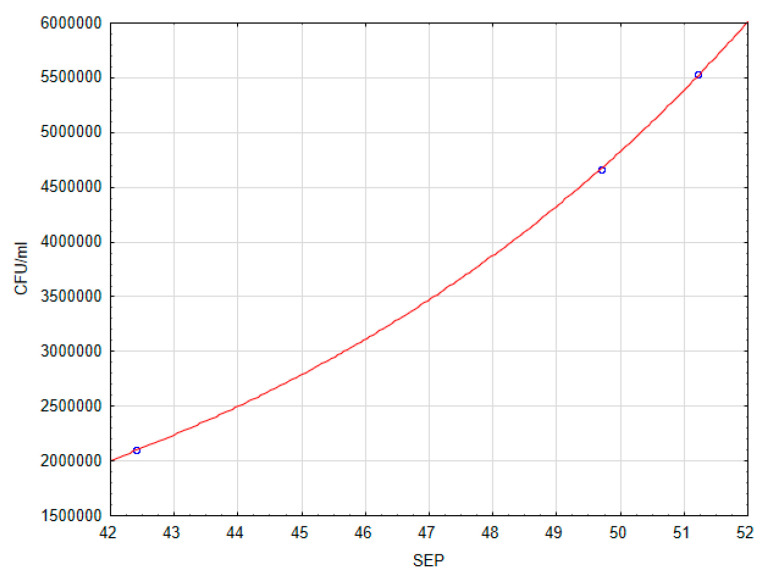
Dependence of the number of CFU/mL on SFE.

The graph of the fitted line is shown in Figure 4.

The authors’ research has shown that there is a correlation between the quantity of adhering bacteria and SFE. The quantity of adhering bacteria increased as the SFE value increased. Some reports available in the literature also emphasized that surfaces with higher SFE were associated with more biofilm formation than surfaces with low SFE [40,41]. Yuan et al. [42] raised an important issue, namely they pointed out that the effect of SFE on bacterial adhesion should be interpreted with great caution, because it can be disturbed by the dominant role of R_a_, especially above 0.06.

Generally recognized hypothesis relating to the influence of surface topography on bacterial attachment currently states that surface protrusions whose sizes are slightly smaller than the bacterial cell size are most effective at reducing the contact area between the cell and substrate by creating a “fakir effect” and thus an unfavorable environment for proliferation [43]. *S. epidermidis* adhere not only to smooth samples with low roughness but also to surfaces that have topographical features greater than the size of bacteria (1–2 μm) [11,44]. A. Cunha et al. [11] indicate that areas in the microscale facilitate bacterial adhesion, providing a large contact area between the cell and the surface. Microstructured surface topography makes it possible for bacterial cells to penetrate spaces between topographic features [9,28]. In the case of topographical features with dimensions smaller than the size of bacteria, the bacteria do not have access to them, because the size of individual traits and the average distance between them prevents the penetration of bacteria [9]. Based on the conducted research and available literature reports, it can be concluded that in order to reduce the adhesion of bacteria, not only superhydrophobicity, but also nano-textures should be produced. Direct laser interference patterning has been shown to be an effective approach for producing periodic surface structures on stainless steel suitable for limiting bacterial retention (up to 99.8% for *E. coli* and 70.6% for *S. aureus*) [45]. Romoli et al. [46] showed that nanosecond pulsed laser irradiation has been found to be an effective approach for reducing *E. coli* adhesion on 316L stainless steel surfaces where the laser scanning strategy and pulse fluence are chosen to achieve homogeneous coverage of the surface with ablation features of limited depth (≤4 µm). To sum up, topography, surface roughness, and SFE are key factors influencing initial bacterial adhesion and biofilm formation [1,9,47].

## 5. Conclusions

The study found that the parameters of the texture (depth, density) influence the SFE value, but the formation of biofilm influenced only the density. Based on the analysis, it can be concluded that there is a correlation between the number of adhering microorganisms and SFE, and surfaces characterized by a high SFE value are more easily colonized by bacteria. 

## Figures and Tables

**Table 1:** SFE values for individual samples.

Density [%]	Depth [µm]	SFE [mJ/m^2^]
15.86	15.69	42.33
44.14	15.69	46.47
10.00	41.50	47.17
30.00	5.00	48.97
15.86	67.31	49.30
30.00	41.50	50.60
30.00	78.00	53.07
44.14	67.31	53.50
50.00	41.50	58.57

**Table 2:** The number of CFU/mL of *S. epidermidis* ATCC 12228 forming biofilm on individual samples.

Density [%]	Depth [µm]	CFU/mL	*OD*
50	5	5.53 × 10^6^	0.437
10	5	2.10 × 10^6^	0.336
10	78	4.66 × 10^6^	0.317

## Data Availability

Not applicable.
